# Immunological Aspects of AXL/GAS‐6 in the Context of Human Liver Regeneration

**DOI:** 10.1002/hep4.1832

**Published:** 2021-12-24

**Authors:** Gregor Ortmayr, Laura Brunnthaler, David Pereyra, Heidemarie Huber, Jonas Santol, Benedikt Rumpf, Sina Najarnia, Rory Smoot, Daphni Ammon, Thomas Sorz, Fabian Fritsch, Michael Schodl, Astrid Voill‐Glaninger, Barbara Weitmayr, Manuela Födinger, Martin Klimpfinger, Thomas Gruenberger, Alice Assinger, Wolfgang Mikulits, Patrick Starlinger

**Affiliations:** ^1^ Department of Surgery Medical University of Vienna General Hospital Vienna Austria; ^2^ Center of Physiology and Pharmacology Institute of Vascular Biology and Thrombosis Research Medical University of Vienna Vienna Austria; ^3^ Department of Medicine I Institute of Cancer Research Comprehensive Cancer Center Medical University of Vienna Vienna Austria; ^4^ Department of Surgery Mayo Clinic Rochester MN USA; ^5^ Department of Laboratory Medicine Viennese Health Network, Clinic Landstraße Vienna Austria; ^6^ Department of Pathology Viennese Health Network, Clinic Landstraße Vienna Austria; ^7^ Department of Laboratory Medicine Viennese Health Network Clinic Favoriten Vienna Austria; ^8^ Department of Pathology Viennese Health Network Clinic Favoriten Vienna Austria; ^9^ Department of Surgery HPB Center, Viennese Health Network, Clinic Favoriten and Sigmund Freud Private University Vienna Austria

## Abstract

AXL and its corresponding ligand growth arrest–specific 6 (GAS‐6) are critically involved in hepatic immunomodulation and regenerative processes. Pleiotropic inhibitory effects on innate inflammatory responses might essentially involve the shift of macrophage phenotype from a pro‐inflammatory M1 to an anti‐inflammatory M2. We aimed to assess the relevance of the AXL/GAS‐6‐pathway in human liver regeneration and, consequently, its association with clinical outcome after hepatic resection. Soluble AXL (sAXL) and GAS‐6 levels were analyzed at preoperative and postoperative stages in 154 patients undergoing partial hepatectomy and correlated with clinical outcome. Perioperative dynamics of interleukin (IL)‐6, soluble tyrosine‐protein kinase MER (sMerTK), soluble CD163 (sCD163), and cytokeratin (CK) 18 were assessed to reflect pathophysiological processes. Preoperatively elevated sAXL and GAS‐6 levels predicted postoperative liver dysfunction (area under the curve = 0.721 and 0.722; *P* < 0.005) and worse clinical outcome. These patients failed to respond with an immediate increase of sAXL and GAS‐6 upon induction of liver regeneration. Abolished AXL pathway response resulted in a restricted increase of sCD163, suggesting a disrupted phenotypical switch to regeneratory M2 macrophages. No association with sMerTK was observed. Concomitantly, a distinct association of IL‐6 levels with an absent increase of AXL/GAS‐6 signaling indicated pronounced postoperative inflammation. This was further supported by increased intrahepatic secondary necrosis as reflected by CK18M65. sAXL and GAS‐6 represent not only potent and easily accessible preoperative biomarkers for the postoperative outcome but also AXL/GAS‐6 signaling might be of critical relevance in human liver regeneration. Refractory AXL/GAS‐6 signaling, due to chronic overactivation/stimulation in the context of underlying liver disease, appears to abolish their immediate release following induction of liver regeneration, causing overwhelming immune activation, presumably via intrahepatic immune regulation.

## Graphical abstract

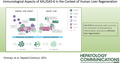

**Abbreviations**
ALP: alkaline phosphatase
ALT: alanine aminotransferase
AST: aspartate aminotransferase
AUC: area under the curve
CD163: cluster of differentiation 163
CLD: chronic liver disease
CK18: cytokeratin 18
cCK18: caspase‐cleaved cytokeratin 18
GAS‐6: growth arrest–specific 6
GGT: gamma‐glutamyl transferase
ICU: intensive care unit
IL: interleukin
IFN: interferon
LD: liver dysfunction
POD: postoperative days
PREOP: preoperative
ROC: receiver operating characteristic
sAXL: soluble Axl
sMerTK: soluble tyrosine‐protein kinase MER
SOCS1/3: suppressor of cytokine signaling 1/3
TAM: acronym Tyro3, Axl and MerTK receptor tyrosine kinases
TGF‐β: transforming growth factor beta
TNF‐α: tumor necrosis factor alpha

The human liver is an organ with remarkable regenerative capacity. Nevertheless, specifically in the context of preexisting liver disease, liver resection might exceed the regenerative reserve. Accordingly, the extent of resection and preexisting functional hepatic impairment have to be carefully assessed, as both affect postoperative recovery.^(^ ^1^, ^2^ ^)^ Especially in humans, in whom underlying liver disease might further affect regenerative processes, liver regeneration is still only incompletely understood.^(^ ^3^ ^)^ Postoperative liver dysfunction (LD), as clinical correlate of impaired regeneration, remains a frequently observed complication of resection as well as a primary determinant of postoperative mortality.^(^ ^4^ ^)^ As there are currently only limited treatment options for patients who develop postoperative LD, prevention via the estimation of patients’ individual risk and subsequent adaptation of surgical strategy are of utmost importance.

Liver regeneration relies on a well‐coordinated interplay of various components that might be disrupted in patients with underlying liver disease. In healthy livers, macrophages were shown to be of importance in a precisely regulated inflammatory response.^(^ ^3^, ^5^ ^)^ Accordingly, macrophage dysfunction could be noted in a variety of chronic liver diseases (CLDs).^(^ ^6^ ^)^ Also, in the context of liver regeneration, macrophages are of distinct relevance, as the depletion of Kupffer cells is associated with delayed liver regeneration in mice.^(^ ^7^, ^8^ ^)^ As resident macrophages, they not only immediately release cytokines relevant for liver regeneration (e.g., interleukin 6 [IL‐6], tumor necrosis factor alpha [TNF‐α]) but were shown to be a guarantee of an orderly inflammatory process.^(^ ^9^ ^)^

However, Kupffer cells exhibit tremendous plasticity, depending on the local metabolic and immune environment. A shift in macrophage polarization, from a pro‐inflammatory M1 to a pro‐regeneratory M2 phenotype, has been well established.^(^ ^10^ ^)^ These phenotypical changes appear to be essential during regenerative processes. In particular, immediately following tissue injury, pro‐inflammatory M1 macrophages are prevailing. Throughout regeneration, though, M1/M2 balance shifts, making pro‐regenerative M2 macrophages the predominant subtype.^(^ ^11^ ^)^ M2 polarization not only dampens pro‐inflammatory M1 responses but also promotes tissue repair.^(^ ^10^, ^11^, ^12^ ^)^ Indeed, missing intrahepatic M2 polarization has been associated with prolonged inflammation and reduced regeneration after liver injury.^(^ ^13^ ^)^ Although M1/M2 dichotomy provides a conceptual framework for our understanding of macrophages and their ambivalent role in the setting of injury, their way of orchestrating inflammation and its resolution is still incompletely understood and might be very dynamic, specifically in the setting of underlying liver disease.^(^ ^14^ ^)^

In this context, TAM receptors (Tyro3, Axl, and Mer receptor tyrosine kinases) and corresponding ligands (GAS‐6 [growth arrest–specific 6], protein S) have been shown to be critically involved in hepatic immunomodulation and further are up‐regulated in patients with liver disease.^(^ ^15^, ^16^, ^17^ ^)^ Accumulating evidence supports rather pleiotropic inhibitory effects on innate inflammatory responses.^(^ ^18^, ^19^, ^20^ ^)^ Interestingly, TAM receptors are also involved in macrophage polarization. Mediating the engulfment of apoptotic bodies, they promote macrophage M2 shift, a process critically disrupted in patients with CLD.^(^ ^17^, ^21^, ^22^ ^)^ In accordance, the hepatic immunological balance is disturbed in the absence of TAM signaling, as an excess of inflammatory cytokines indicated pronounced inflammation due to insufficient inhibition of innate inflammatory responses.^(^ ^23^, ^24^ ^)^ In mice, deficient TAM signaling was linked to pronounced inflammation and delayed liver regeneration following acute liver injury.^(^ ^23^, ^24^ ^)^ Furthermore, mice with a deficiency of either AXL or GAS‐6 suffered from impaired regeneration with a much higher incidence than wild‐type animals.^(^ ^23^, ^24^ ^)^

Given the established association of TAM signaling and CLD, as well as its potential relevance in liver regeneration, we aimed to (1) explore whether preoperative soluble Axl (sAXL) and GAS‐6 levels could predict postoperative outcome after liver resection, (2) identify potential differences in their perioperative dynamics in patients with and without insufficient postoperative liver regeneration, and (3) gain mechanistic insight into how TAM signaling might affect liver regeneration in humans, particularly on the background of underlying liver disease.

## Patients and Methods

For this study, a total of 154 patients were recruited at two different institutions, comprising the General Hospital of Vienna/Medical University of Vienna (Austria) and the hospital Klinikum Favoriten (Austria). Patients undergoing liver resection were followed prospectively over a postoperative period of 90 days. sAXL, GAS‐6, and soluble tyrosine‐protein kinase MER (sMerTK) as well as soluble cluster of differentiation 163 (sCD163), IL‐6, and cytokeratin 18 (CK18) were evaluated within the immediate perioperative period (1 day before surgery [PREOP] and 1 day [POD1] as well as 5 days afterward [POD5]. Furthermore, in a subset of 63 patients, intraoperative samples were obtained (see Supporting Methods) to evaluate alterations in the early phase of liver regeneration.

Essential patient‐related data were assessed as listed in Table 1. The extent of resection was classified as minor or major resection (< 3 segments = minor; ≥ 3 segments = major) according to the IHPBA‐Brisbane‐2000 nomenclature.^(^ ^25^ ^)^ Postoperative outcome was prospectively documented and classified in LD (International Study Group of Liver Surgery criteria), postoperative morbidity (Calvien‐Dindo classification), and postoperative mortality.^(^ ^26^, ^27^ ^)^ For more details, refer to the Supporting Methods section.

**TABLE 1:** Demographics of Entire Cohort

	Cohort Total (n = 154)	Cohort AXL_low_ (n = 70)	Cohort AXL_high_ (n = 80)		Cohort GAS‐6_low_ (n = 99)	Cohort GAS‐6_high_ (n = 44)	
			n (%) – Median [Range]				
Age (years)	64.2 (22.16‐89.31)	61.7 (31.7‐89.3)	65.2 (22.2‐86.1)	[0.154]	64.1 (22.16‐89.31)	65.2 (35.61‐84.68)	[0.112]
Sex							
Male	105 (68.2)	43 (61.4)	60 (75.0)	[0.074]	70 (70.7)	32 (72.7)	[0.805]
Female	49 (31.8)	27 (38.6)	20 (25.0)	[0.074]	29 (29.3)	12 (27.3)	[0.805]
Neoplastic entity							
mCRC	62 (40.3)	49 (70.0)	13 (16.2)	<0.005	57 (57.6)	5 (11.4)	<0.005
HCC	50 (32.5)	4 (5.7)	44 (55.0)	<0.005	20 (20.2)	28 (63.3)	<0.005
CCC	25 (16.2)	7 (10.0)	16 (20.0)	[0.09]	12 (12.1)	10 (22.7)	[0.105]
Non‐neoplastic	12 (7.8)	8 (11.4)	4 (5.0)	[0.148]	7 (7.1)	0 (0.0)	[0.1]
Other	5 (3.2)	2 (2.9)	3 (3.8)	[1.0]	3 (3.0)	1 (2.3)	[0.8]
Resection extent							
Major	98 (63.6)	46 (65.7)	49 (61.2)	[0.571]	64 (64.6)	28 (63.6)	[0.907]
Minor	56 (36.4)	24 (34.3)	31 (38.8)	[0.571]	35 (35.4)	16 (36.4)	[0.907]
Outcome							
Postoperative LD—yes	18 (11.7)	1 (1.7)	15 (21.7)	<0.005	4 (4.8)	12 (30.8)	<0.005
Morbidity—yes	77 (50.0)	26 (37.1)	47 (58.8)	[0.008]	36 (36.4)	33 (75.0)	<0.005
Severe morbidity—yes	40 (26.0)	13 (18.6)	25 (31.3)	[0.075]	15 (15.2)	21 (47.7)	<0.005
90‐day mortality—yes	5 (3.2)	0 (0.0)	4 (5.3)	[0.123]	0 (0.0)	4 (9.8)	<0.005
ICU days	1.0 (0‐26)	1.0 (0‐5)	1.0 (0‐26)	[0.123]	1.0 (0‐10)	2.0 (0‐26)	[0.014]
Hospitalization days	8.0 (3‐117)	8.0 (4‐61)	10.0 (3‐117)	[0.53]	8.0 (3‐61)	12.5 (4‐117)	<0.005
Liver histology							
Fibrosis—yes	112 (72.7)	46 (73.0)	63 (81.8)	[0.212]	71 (74.7)	34 (82.9)	[0.296]
Grade 0‐II	77 (75.6)	57 (90.5)	51 (66.2)	<0.005	82 (86.3)	23 (56.1)	<0.005
Grade III‐IV	35 (24.5)	6 (9.5)	26 (33.8)	<0.005	13 (13.7)	18 (43.9)	<0.005
NASH—yes	52 (33.8)	26 (49.1)	26 (40.0)	[0.324]	38 (52.1)	13 (31.7)	[0.036]
CASH—yes	29 (18.8)	20 (40.8)	9 (15.5)	<0.005	25 (36.8)	3 (8.3)	<0.005
SOS—yes	13 (8.4)	10 (16.4)	3 (4.3)	[0.021]	13 (14.6)	0 (0.0)	[0.009]
Steatosis (%)	10.0 (0.0‐100.0)	5.0 (0.0‐100.0)	10.0 (0.0‐80.0)	[0.62]	7.5 (0.0‐85)	12.5 (0.0‐100.0)	[0.88]
Preoperative parameters							
PT (%)	101.0 (40.0‐150.0)	103.0 (45.0‐150.0)	97.0 (40.0‐150.0)	[0.071]	103.0 (45.0‐150.0)	93.0 (40.0‐150.0)	<0.005
ALP (U/L)	90.0 (38.0‐707.0)	90.0 (38.0‐418.0)	90.5 (42.0‐423.0)	[0.32]	85.0 (38.0‐418.0)	104.0 (48.0‐423.0)	<0.005
GGT (U/L)	68.0 (11.0‐1576.0)	48.5 (11.0‐505.0)	92.0 (13.0‐1335.0)	<0.005	50.0 (11.0‐710.0)	157.0 (13.0‐1,335.0)	<0.005
AST (U/L)	31.0 (17.0‐224.0)	29.0 (17.0‐71.0)	34.0 (17.0‐224.0)	[0.024]	28.0 (17.0‐113.0)	52.0 (21.0‐224.0)	<0.005
ALT (U/L)	31.0 (7.0‐372.0)	26.0 (7.0‐81.0)	35.0 (8.0‐372.0)	[0.037]	25.0 (7.0‐123.0)	46.5 (8.0‐372.0)	<0.005
Albumin (g/L)	42.0 (30.2‐50.0)	42.75 (34.0‐50.0)	42.0 (30.2‐47.6)	[0.031]	42.0 (34.0‐50.0)	40.1 (30.2‐47.6)	[0.018]
Bilirubin (mg/dL)	0.6 (0.1‐6.64)	0.52 (0.0‐2.87)	0.66 (0.0‐6.64)	<0.005	0.55 (0.0‐3.17)	0.81 (0.0‐6.64)	[0.025

Abbreviations: 95% CI, 95% confidence interval; OR, odds ratio; SB, serum bilirubin.mCRC, metastatic colorectal cancer; HCC, hepatocellular cancer; CCC, cholangiocellular cancer; ICU, intensive care unit; NASH, non‐alcoholic steatohepatitis; CASH, chemotherapy associated steatohepatitis; SOS, sinusoidal obstruction syndrome; PT, prothrombin time; ALP, alkaline phosphatase; GGT, gamma‐glutamyl transpeptidase; AST, aspartate aminotransferase; ALT, alanine aminotransferasase.

The study was conducted in adherence to the Declaration of Helsinki and was approved by the Ethics Committee of the Medical University of Vienna. Ahead of participation, informed consent was obtained from all patients (EK Nr. 16‐253‐0117 and EK 14‐122‐0714).

### Measurement of sAXL/GAS‐6, sMerTK, sCD163, IL‐6, CK18, and Routine Laboratory Parameters

Perioperative blood parameters of liver function were measured as part of the clinical routine by the local institutional laboratory and were documented prospectively (Table 1). Experimental parameters, including sAXL, sMerTK and GAS‐6, as well as sCD163, IL‐6 and CK18, were determined using enzyme‐linked immunosorbent assays. For a more detailed description of experimental procedures refer to the Supporting Methods section.

### Statistical Analyses

IBM SPSS software version 20.0 (IBM Corp., Armonk, NY) and GraphPad Prism 8 (GraphPad Software, La Jolla, CA) were used for statistical data analyses, which was based on nonparametric testing for either paired or independent samples (Mann‐Whitney U test, Wilcoxon sign test, Spearman‐Rho, or Pearson correlation analysis). For the comparison of prevalence and incidence between the groups, chi‐squared tests were conducted.

The diagnostic validity of experimental parameters for LD was assessed by receiver operating characteristic (ROC) analysis considering the area under the curve (AUC). For preoperative risk stratification, cutoff values based on obtained data points were deduced using the Youden index. Calculation provided optimal cutoff values at concentrations, representing the highest sum of sensitivity and specificity and hence maximum discrimination in distinguishing high‐risk and low‐risk groups. *P* values < 0.05 were considered statistically significant.

## Results

### Patients With sAXL^HIGH^ and GAS‐6^HIGH^ Are at Risk of Postoperative LD and Worse Clinical Outcome

**FIG. 1:**
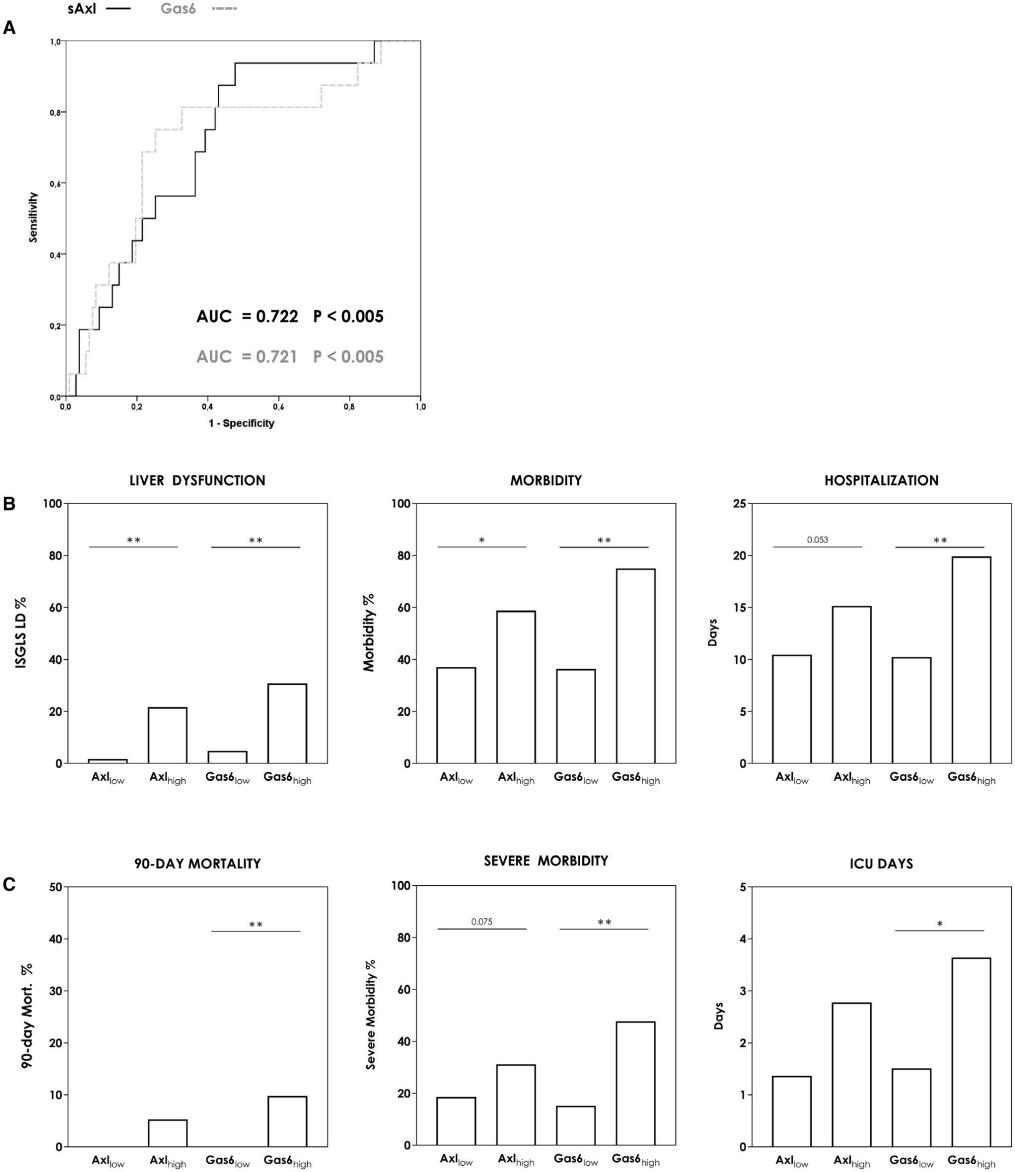
Prediction of postoperative outcome according to sAxl/Gas6 serum concentrations. Preoperative predictive value of sAxl and Gas6 for postoperative LD is demonstrated by ROC analysis (A). Classification according to deduced cutoffs (sAxl = 31.91 ng/mL; Gas6 = 34.42 mg/mL) demonstrates the variance of incidence among low‐risk and high‐risk subgroups for postoperative LD and morbidity (chi‐squared test) (B) as well as 90‐day mortality and severe postoperative morbidity (chi‐squared test) (C). Differences in postoperative and ICU stay are shown in (B) and (D) (two‐tailed unpaired Student *t* test). *P < 0.05; **P < 0.005. Abbreviation: ISGLS, International Study Group of Liver Surgery.

Initially, we evaluated the predictive potential of preoperative sAXL and GAS‐6 serum concentrations in patients undergoing liver resection. ROC analysis revealed distinct validity as preoperative markers for postoperative LD with an area under the curve (AUC) of 0.722 (sAXL, *P* < 0.005) and 0.721 (GAS‐6, *P* < 0.005) (Fig. 1A). Furthermore, their similarity in prediction as well as their functional association indicated significant correlation, which could be verified as illustrated in Supporting Fig. S2A. (*R* = 0.707, *P* < 0.005).

For improved risk stratification, we defined cutoff values according to respective ROC analysis (sAXL = 31.91 ng/mL; GAS‐6 = 34.42 mg/mL), enabling the most accurate identification of patients with high risk of postoperative LD (sAXL: sensitivity = 93.8%, specificity = 52.3%; GAS‐6: sensitivity = 75.0%, specificity = 74.8%). Patients with elevated GAS‐6 and sAXL levels were found to significantly differ in baseline characteristics (Table 1) and displayed a significantly higher frequency of cirrhosis and elevated liver function/damage parameters. Furthermore, in terms of postoperative outcome, the incidence of postoperative LD (sAXL: 1.67%‐21.74% [*P* < 0.005]; GAS‐6: 4.76%‐30.76% [*P* < 0.005]) and morbidity (sAXL: 37.14%‐58.75% [*P* = 0.008]; GAS‐6: 36.36%‐75.00% [*P* < 0.005]) was significantly increased in patients surpassing our cutoff values (Fig. 1B and Supporting Fig. S2B). In line with these findings, we observed a higher incidence of severe morbidity (sAXL: *P* = 0.075; GAS‐6: *P* < 0.005) and postoperative 90‐day mortality (sAXL: *P* = 0.123; GAS‐6: *P* = 0.007) in patients exceeding our preoperative sAXL and GAS‐6 cutoff values (Fig. 1C). These patients further displayed prolonged intensive care unit (ICU) (sAXL: *P* = 0.123; GAS‐6: *P* = 0.014) and hospital stay (sAXL: *P* = 0.053; GAS‐6: *P* < 0.005) (Fig. 1B,C).

When examining the prognostic value of sAXL and GAS‐6 levels in patients undergoing major liver resection, being of higher risk to develop postoperative complications, the trends were similar to the entire cohort. Obtained preoperative predictive validity was equal (sAXL AUC = 0.726 [*P* = 0.009] and GAS‐6 AUC = 0.726 [*P* = 0.009]) (Supporting Fig. S3A). Risk stratification, according to the prior chosen cutoffs, again demonstrated a significantly increased incidence of postoperative LD and morbidity within the high‐risk groups (LD: sAXL, 2.7%‐31.70% [*P* < 0.005]; GAS‐6, 7.69%‐41.67% [*P* < 0.005]; morbidity: sAXL, 43.48%‐63.27% [*P* = 0.053]; GAS‐6, 42.19%‐82.14% [*P* < 0.005]) (Supporting Fig. S3A). Preoperative sAXL and GAS‐6 concentrations were consequently consistent in the prediction of postoperative outcome regardless of further influencing/prognostic factors, except advanced fibrosis and cirrhosis, as sAXL performance appeared to be limited in these patients (low [fibrosis grade 0‐2]: sAXL, AUC = 0.763 [*P* = 0.005]; advanced [fibrosis grade 3‐4]: sAXL, AUC = 0.568 [*P* = 0.67]) (Supporting Fig. S3B). For GAS‐6, neither the degree of fibrosis (low [fibrosis grade 0‐2]: GAS‐6, AUC = 0.688 [*P* = 0.043]; advanced [fibrosis grade 3‐4]: GAS‐6, AUC = 0.795 [*P* = 0.065]) (Supporting Fig. S3B), nor resection extent strikingly altered the predictive value. For validation of their independence in the prediction of postoperative LD, a multivariate analysis was performed. Only sAXL and GAS‐6, as well as the extent of hepatic resection, remained significant predictors of postoperative LD. The results from the final model fit are given in Table 3. These data suggest that preoperative sAXL, except for advanced fibrosis, and GAS6 can predict postoperative outcomes regardless of the extent of resection and underlying grade of fibrosis. Unfortunately, the development of a model‐based prediction combining the benefits of both markers did not yield improved outcome prediction as visualized in Supporting Table S1. Limiting might be the strong correlation observed between sAXL and GAS6 (R = 0.707, *P* < 0.005) (Supporting Fig. S2A).

### sAXL and GAS‐6 Dynamics Following Partial Hepatectomy

**FIG. 2:**
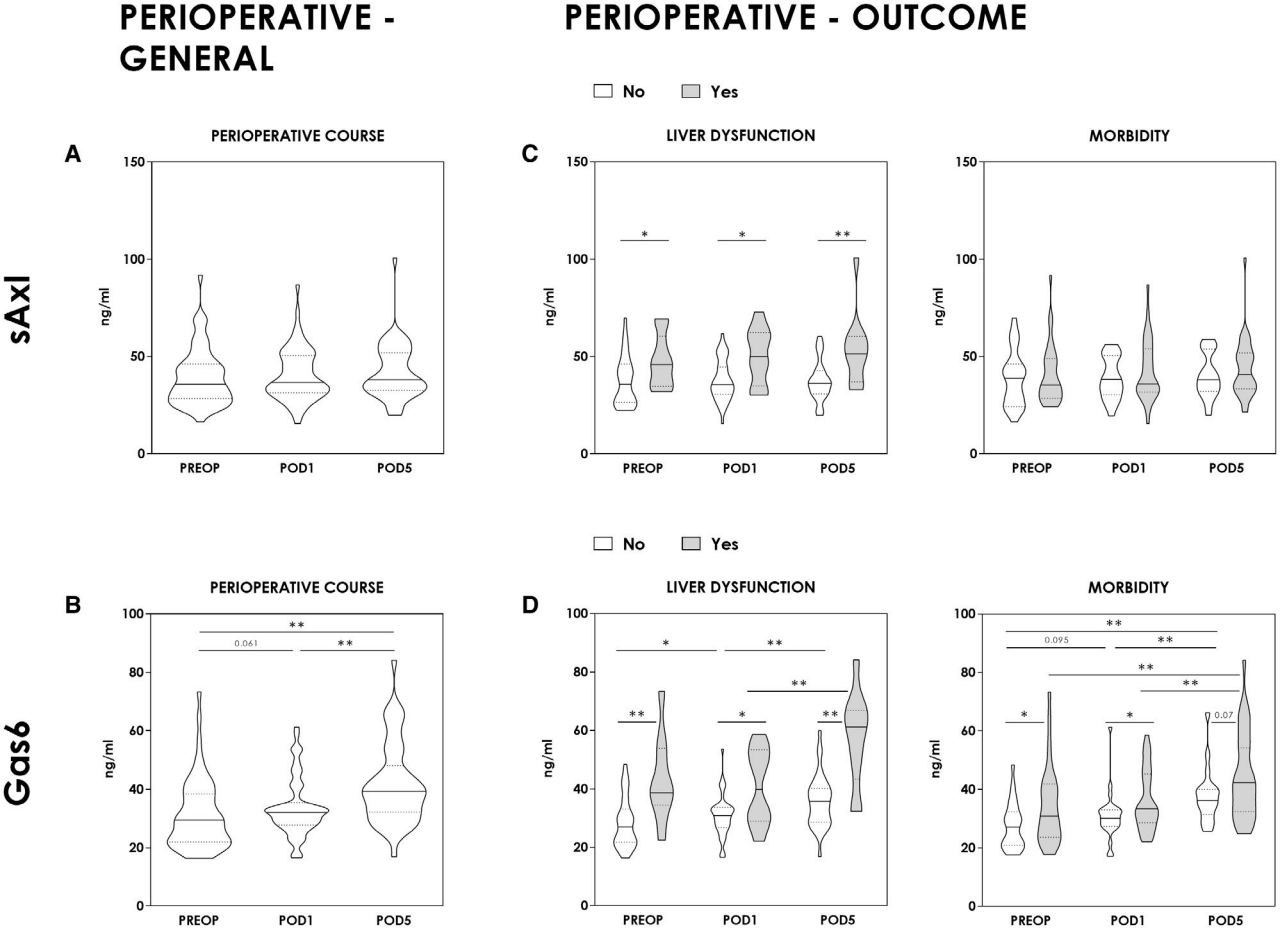
Perioperative time course. sAxl and Gas6 concentrations were measured preoperatively (PREOP), on postoperative day 1 (POD1), and postoperative day 5 (POD5). Perioperative dynamic is illustrated in general (Mann‐Whitney *U* test and Wilcoxon signed‐rank test) (A,B) as well as separately for LD and postoperative morbidity (Mann‐Whitney U test and Wilcoxon signed‐rank test) (C,D). *P < 0.05; **P < 0.005.

Murine models indicate a central role of the receptor‐ligand pair AXL/GAS‐6 in liver regeneration. However, the perioperative dynamics in humans have never been evaluated. Given the significant association of sAXL/GAS‐6 with liver functional outcomes, we aimed to explore the dynamics of AXL/GAS‐6 levels in patients undergoing liver resection. In a subset of 63 patients (for baseline characteristics of the perioperative cohort refer Table 2), we therefore evaluated perioperative sAXL and GAS‐6 dynamics and could observe a steady rise of GAS‐6 serum level from preOP until POD5, while sAXL levels remained fairly stable (Fig. 2A,B).

**TABLE 2:** Demographics of PeriOP Cohort

	Cohort Total (n = 63)	Cohort AXL_low_ (n = 23)	Cohort AXL_high_ (n = 38)		Cohort GAS‐6_low_ (n = 40)	Cohort GAS‐6_high_ (n = 18)	
			n (%) – Median [Range]				
Age (years)	63.29 (24.33‐89.31)	60.47 (31.97‐89.21)	65.06 (37.18‐86.14)	[0.326]	61.27 (31.97‐89.21)	67.72 (46.9‐81.21)	[0.305]
Sex							
Male	41 (65.1)	14 (60.9)	26 (68.4)	[0.547]	26 (65.0)	11 (61.1)	[0.776]
Female	22 (34.9)	9 (39.1)	12 (31.6)	[0.547]	14 (35.0)	7 (38.9)	[0.776]
Neoplastic entity							
mCRC	10 (15.9)	8 (34.8)	2 (5.3)	<0.005	8 (20.0)	2 (11.1)	[0.708]
HCC	22 (34.9)	2 (8.7)	19 (50.0)	<0.005	11 (27.5)	9 (50.0)	[0.095]
CCC	22 (34.9)	7 (30.4)	14 (36.8)	[0.61]	12 (30.0)	7 (38.9)	[0.505]
Non‐neoplastic	7 (11.1)	5 (21.7)	2 (5.3)	[0.093]	7 (17.5)	0 (0.0)	[0.087]
Other	2 (2.3)	1 (4.3)	1 (2.6)	[1.0]	2 (5.0)	0 (0.0)	[1.0]
Resection extent							
Major	52 (82.5)	21 (91.3)	29 (76.3)	[0.182]	34 (85.0)	15 (83.3)	[1.0]
Minor	11 (17.5)	2 (8.7)	9 (23.7)	[0.182]	6 (15.0)	3 (16.7)	[1.0]
Outcome							
Postoperative LD—yes	11 (20.0)	0 (0.0)	10 (30.3)	[0.008]	2 (5.9)	7 (43.8)	<0.005
Morbidity—yes	34 (54.0)	10 (43.5)	22(57.9)	[0.275]	18 (45.0)	13 (72.2)	[0.055]
Severe morbidity—yes	18 (28.6)	4 (17.4)	13 (34.2)	[0.156]	7 (17.5)	9 (50.0)	[0.024]
90‐day mortality—yes	1 (1.8)	0 (0.0)	1 (2.9)	[1.0]	0 (0.0)	1 (5.9)	[0.321]
ICU days	1.0 (0.0‐26.0)	1.0 (0.0‐5.0)	1.0 (0.0‐26.0)	[0.045]	1.0 (0.0‐10.0)	2.0 (0.0‐26.0)	[0.029]
Hospitalization days	10.0 (3.0‐117.0)	8.0 (4.0‐46.0)	11.5 (3.0‐117.0)	[0.512]	8.0 (3.0‐46.0)	14.0 (5.0‐ 117.0)	[0.016]
Liver histology							
Fibrosis—yes	42 (75.0)	13 (68.4)	28 (77.8)	[0.522]	26 (70.3)	12 (80.0)	[0.731]
Grade 0‐II	41 (73.2)	16 (84.2)	25 (69.4)	[0.334]	31 (83.3)	10 (66.7)	[0.26]
Grade III‐IV	15 (26.8)	3 (15.8)	11 (30.6)	[0.334]	6 (16.2)	5 (33.3)	[0.26]
NASH—yes	19 (43.2)	9 (64.3)	10 (35.7)	[0.079]	12 (52.2)	5 (31.2)	[0.195]
CASH—yes	7 (18.9)	6 (46.2)	1 (4.5)	[0.006]	6 (28.6)	0 (0.0)	[0.071]
SOS—yes	3 (5.7)	1 (5.0)	2 (6.5)	[1.0]	3 (8.6)	0 (0.0)	[0.378]
Steatosis (%)	5.0 (0.0‐100.0)	5.0 (0.0‐100.0)	10.0 (0.0‐80.0)	[0.634]	5.0 (0.0‐85.0)	17.5 (0.0‐80.9)	[0.361]
Preoperative parameters							
PT (%)	101.0 (62.0‐150.0)	106.0 (83.0‐136.0)	99.0 (62.0‐150.0)	[0.275]	104.0 (80.0‐137.0)	94.0 (62.0‐150.0)	[0.05]
ALP (U/L)	89.0 (38.0‐423.0)	98.0 (38.0‐169.0)	88.0 (42.0‐423.0)	[0.731]	86.5 (38.0‐230.0)	90.5 (51.0‐423.0)	[0.353]
GGT (U/L)	67.0 (11.0‐710.0)	54.0 (11.0‐505.0)	82.0 (13.0‐710.0)	[0.354]	54.0 (11.0‐710.0)	142.0 (13.0‐562.0)	[0.044]
AST (U/L)	28.0 (17.0‐208.0)	27.0 (19.0‐51.0)	30.0 (17.0‐208.0)	[0.43]	27.0 (17.0‐113.0)	45.0 (22.0‐208.0)	<0.005
ALT (U/L)	27.0 (9.0‐372.0)	26.0 (11.0‐78.0)	27.0 (9.0‐372.0)	[0.523]	25.0 (10.0‐123.0)	42.0 (9.0‐372.0)	[0.017]
Albumin (g/L)	42.0 (30.2‐48.0)	43.5 (38.0‐48.0)	42.0 (30.2‐47.6)	[0.06]	42.0 (36.0‐48.0)	40.0 (30.2‐47.6)	[0.251]
Bilirubin (mg/dL)	0.63 (0.29‐6.64)	0.49 (0.29‐1.09)	0.9 (0.34‐6.64)	<0.005	0.55 (0.29‐3.17)	1.0 (0.42‐6.64)	[0.041]

Abbreviation: 95% CI, 95% confidence interval; ALP, alkaline phosphatase; ALT, alanine aminotransferasase; AST, aspartate aminotransferase; CASH, chemotherapy associated steatohepatitis; CCC, cholangiocellular cancer; GGT, gamma‐glutamyl transpeptidase; HCC, hepatocellular cancer; ICU, intensive care unit; mCRC, metastatic colorectal cancer; NASH, non‐alcoholic steatohepatitis; OR, odds ratio; PT, prothrombin time; SOS, sinusoidal obstruction syndrome.

**TABLE 3:** Multivariable Analysis for LD

Parameter	Univariate Analysis			Multivariable Analysis sAxl			Multivariable Analysis Gas6		
	OR	95% CI	*P* Value	OR	95% CI	*P* Value	OR	95% CI	*P* Value
sAxl (ng/mL)	**1.049**	**1.013‐1.086**	**0.007**	**1.056**	**1.017‐1.096**	**0.005**	—	—	—
Gas6 (ng/mL)	**1.049**	**1.012‐1.088**	**0.010**	—	—	—	**1.062**	**1.020‐‐1.106**	**0.004**
Gender	0.974	0.340‐2.794	0.961						
Neoplastic entity	1.263	0.816‐1.955	0.294						
Hepatic resection	**6.154**	**1.352‐28.011**	**0.019**	**6.423**	**1.307‐31.576**	**0.022**	**7.580**	**1.428‐40.233**	**0.017**
Co‐factors									
Cirrhosis	1.937	0.555‐6.761	0.300						
Fibrosis (≥ III)	1.545	0.491‐4.868	0.457						
CASH	0.429	0.089‐2.078	0.293						
SOS	0.715	0.085‐6.056	0.759						
Steatosis (%)	1.007	0.986‐1.028	0.545						
Preoperative parameters									
Platelets (×10^3^/µL)	0.997	0.990‐1.005	0.450						
Bilirubin (mg/dL)	1.816	0.984‐3.351	0.056						
PT (%)	0.994	0.969‐1.020	0.632						
ALP (U/L)	**1.006**	**1.001‐1.011**	**0.016**	0.999	0.990‐1.008	0.824	0.998	0.990‐1.007	0.704
GGT (U/L)	**1.002**	**1.000‐1.004**	**0.030**	1.000	0.996‐1.004	0.972	1.000	0.996‐1.004	0.957
AST (U/L)	**1.015**	**1.003‐1.027**	**0.013**	0.999	0.982‐1.015	0.880	0.999	0.982‐1.017	0.940
ALT (U/L)	1.004	0.995‐1.012	0.417						
Albumin (g/L)	**0.864**	**0.758‐0.986**	**0.031**	0.896	0.765‐1.050	0.175	0.921	0.786‐1.080	0.311
Age (years)	1.024	0.982‐1.067	0.268						

Statistically significant values are shown in bold.

Abbreviations: 95% CI, 95% confidence interval; ALP, alkaline phosphatase; ALT, alanine aminotransferasase; AST, aspartate aminotransferase; CASH, chemotherapy associated steatohepatitis; GGT, gamma‐glutamyl transpeptidase; OR, odds ratio; PT, prothrombin time; SOS, sinusoidal obstruction syndrome.

Major resections, which require more intensive liver regeneration, showed an even more pronounced dynamic, whereas patients undergoing minor resection were missing a significant increase following liver resection (Supporting Fig. S4).

Most importantly, we could observe outcome‐dependent postoperative discrepancies, in particular for GAS‐6, whereas sAXL concentrations showed only limited postoperative outcome‐related dynamics. In general, measured concentrations were distinctly higher in patients with LD for GAS‐6 (PREOP, *P* < 0.005; POD1, *P* = 0.041; POD5, *P* < 0.005) and sAXL (PREOP, *P* = 0.033; POD1, *P* = 0.016; POD5, *P* = 0.004) (Fig. 2C,D). Furthermore, serum concentrations of GAS‐6 overall rose until POD5, but far more pronounced in patients with postoperative LD (*P* = 0.005) (Fig. 2D). However, the immediate GAS‐6 response in patients with LD appeared to be blunted, as we observed a less pronounced initial release on POD1 than in patients without postoperative LD (increase on POD1 only in patients with no LD [*P* = 0.029]) (Fig. 2D).

As a clinical hallmark of postoperative LD, the evaluation of postoperative morbidity completed our analysis. Trends in postoperative morbidity paralleled the association of GAS‐6 and the incidence of liver dysfunction. Postoperative morbidity was significantly associated with higher GAS‐6 concentrations (PREOP, *P* = 0.02; POD1, *P* = 0.033; POD5, *P* = 0.07) (Fig. 2D). Additionally, the perioperative course showed an initial slight increase until POD1, except for patients with postoperative morbidity (PREOP‐POD1: Morb., *P* = 0.329; No Morb., *P* = 0.095). This was followed by a steep increase in GAS‐6 concentrations peaking at POD5 and, similar to patients with LD, more pronounced in the case of postoperative morbidity (POD1‐POD5: Morb., *P* < 0.005; No Morb., *P* < 0.005) (Fig. 2D). Thus, sAXL/GAS‐6 are not only predictive of postoperative outcomes following liver resection, but also observed postoperative dynamics suggest a distinct pattern of response associated with human liver regeneration.

### Immediate Rise of sAXL and GAS‐6 as Indication of their Contribution to Liver Regeneration

Distinct postoperative dynamics of sAXL and GAS‐6 with regard to the extent of liver resection as well as the fact that patients with LD and morbidity failed to increase on POD1 led us to hypothesize that patients with underlying liver disease might suffer from prior chronic and consequently exhaustive activation of AXL/GAS‐6 signaling, especially because patients with preoperatively elevated concentrations, as found in the context of CLD, were unable to respond with a further boost in the signaling activity after induction of liver regeneration.

**FIG. 3:**
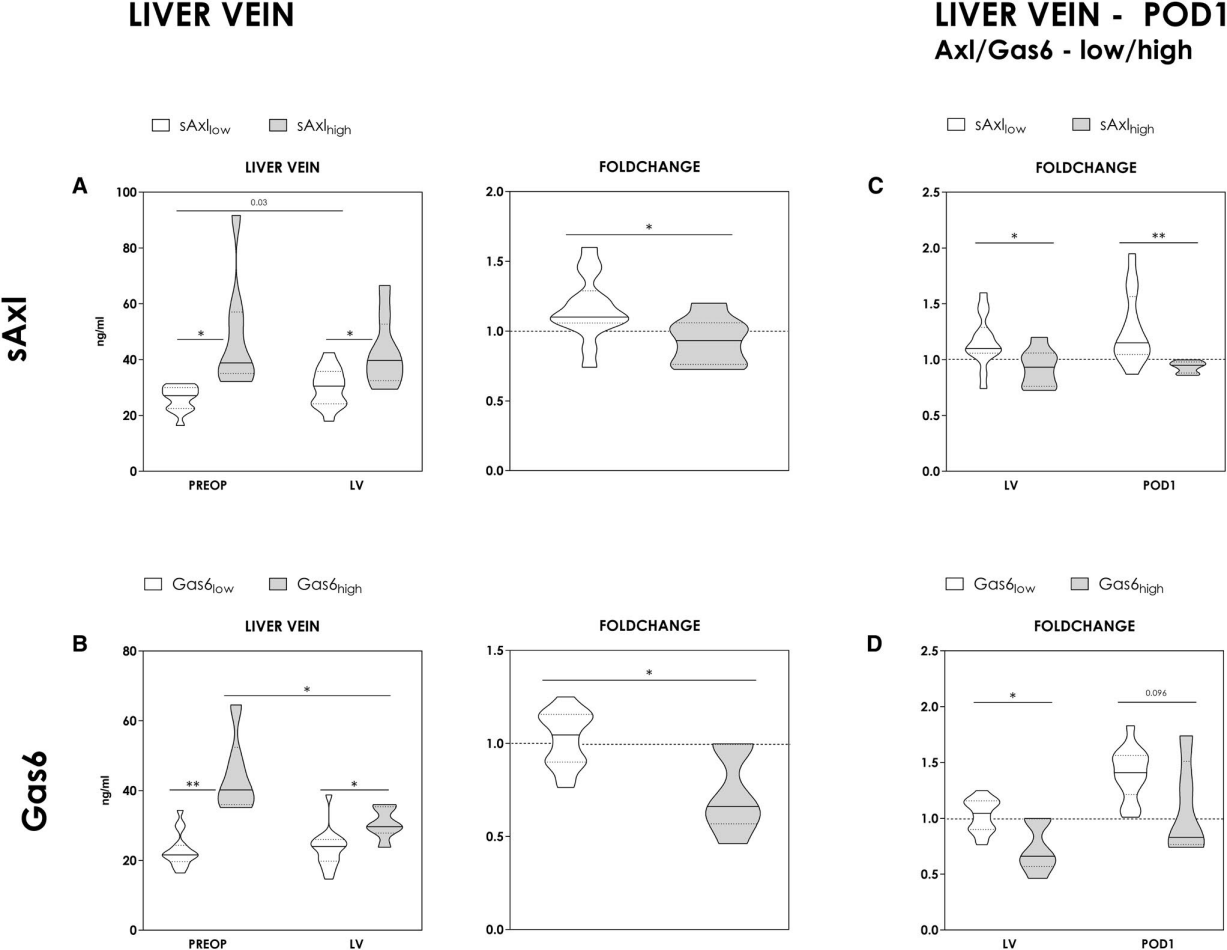
Dynamics of sAxl and Gas6 during early liver regeneration. To evaluate alterations in the early phase, sAxl and Gas6 levels were assessed in the liver vein (LV) of the regenerating liver lobe 2 hours after induction of regeneration and on POD1. Results are shown in relation to the preoperative concentrations to exemplify postresection alterations. Postoperative changes are further indicated by corresponding fold change (concentrations in the LV divided by baseline concentrations [PREOP]). (A,B) Observed dynamics [absolute and fold change] in the LV and on POD1 were compared between high‐risk and low‐risk groups (Mann‐Whitney U test and Wilcoxon signed‐rank test) (A,B & C,D). Classification was based on antecedently chosen cutoff values. (Mann‐Whitney *U* test). *P < 0.05; **P < 0.005.

Consequently, we aimed to investigate circulating sAXL and GAS‐6 dynamics in more detail, namely immediately after induction of liver regeneration. To address alterations in this early period, we analyzed liver vein samples taken 2 hours after induction of liver regeneration. Indeed, we could observe a significant increase of sAXL and GAS‐6 levels immediately following induction of liver regeneration (i.e., 2 hours after portal vein ligation) (Fig. 3). When patients were again classified based on the previously defined cutoff values, we observed consistently higher sAXL and GAS‐6 levels in patients with preexisting elevation of sAXL and GAS‐6 (sAXL, *P* = 0.014 [Fig. 3A]; GAS‐6, *P* = 0.012 [Fig. 3B]). However, again subgroups with high preoperative sAXL and GAS‐6 concentrations did lack an immediate induction during this very early time point in human liver regeneration (Preop–liver vein: sAXL, *P* = 0.263 [Fig. 3A]; GAS‐6, *P* = 0.028 [Fig. 3B]). Overall, serum concentrations even tended to decline in patients with high preoperative sAXL and GAS‐6, whereas patients with no elevation preoperatively remained fairly stable or increased (fold change: sAXL_high_ [preop] vs. sAXL_low_ [preop], *P* = 0.017 [Fig. 3A]; GAS‐6_high_ [preop] vs. GAS‐6_low_ [preop], *P* = 0.012 [Fig. 3B]).

Observed tendencies continued up to POD1, as illustrated in Fig. 3C,D. Serum concentrations of sAXL or GAS‐6 only rose in patients without preoperatively elevated sAXL/GAS‐6 levels (fold change: sAXL_high_ [preop] vs. sAXL_low_ [preop], *P* < 0.005; GAS‐6_high_ [preop] vs. GAS‐6_low_ [preop], *P* = 0.096). These data further support the hypothesis that an acute dynamic response in AXL/GAS‐6 signaling could be required for functional liver regeneration.

### Diminished M2 Polarization in Patients With GAS‐6^HIGH^

As we had further substantiated the observation that patients with exaggerated preoperative AXL/GAS‐6‐signaling appeared to show chronic exhaustion of this immunoregulatory pathway, we also aimed to assess intrahepatic inflammatory changes in more detail. Resident macrophages (Kupffer cells) might be of relevance, as GAS‐6 mediated AXL signaling/clearance of apoptotic cells has been shown to favor a regenerative macrophage phenotype that is essential for the resolution of inflammation.

**FIG. 4:**
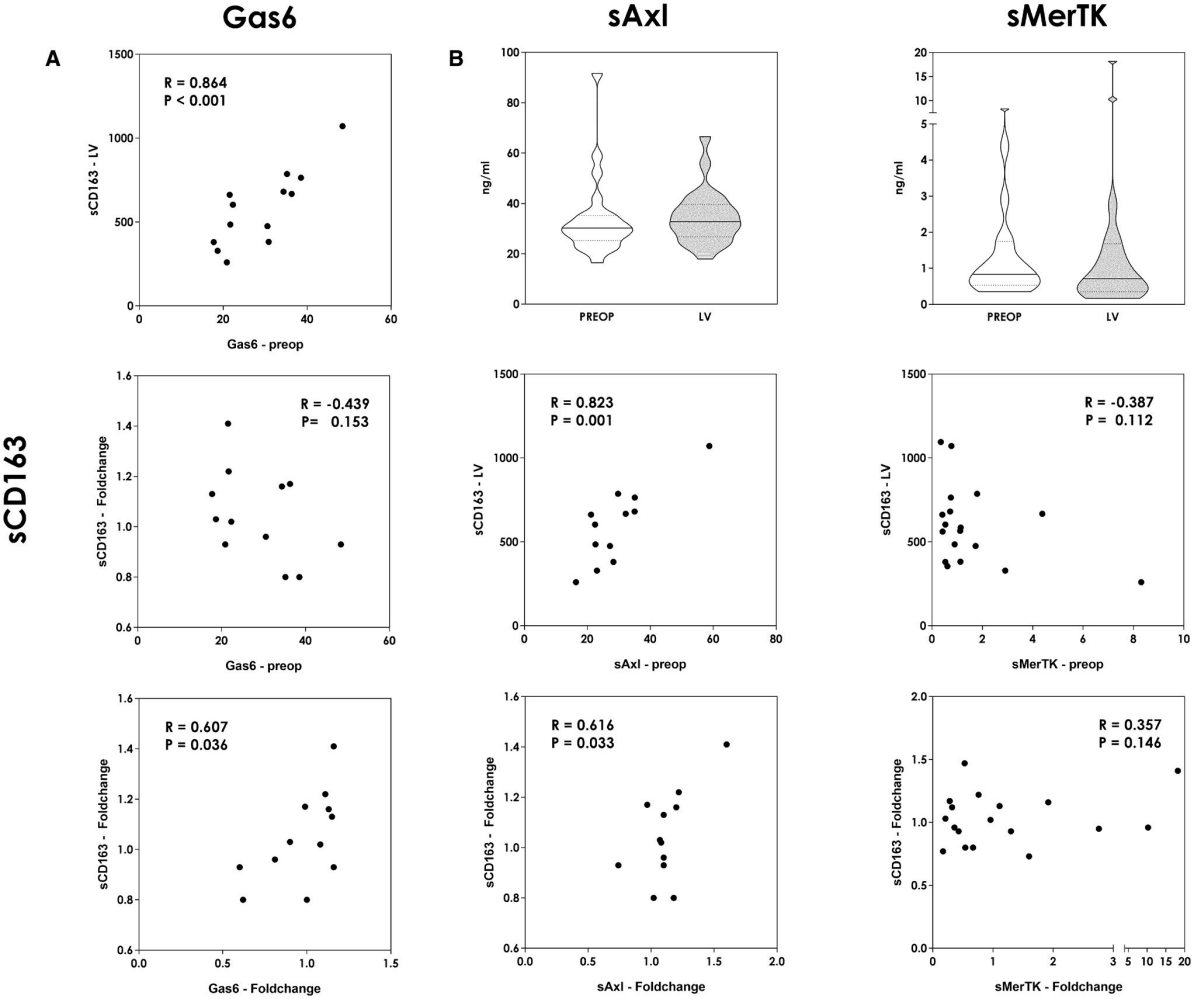
Postoperative M2 polarization. Correlation of Gas6, sAxl, and MerTK concentrations with sCD163. Analyses were performed for absolute values (in nanograms per milliliter [ng/mL]) as well as the postoperative fold change (LV divided by baseline concentrations [PREOP]) (A,B). sAxl as well as sMerTK concentrations are shown for the immediate perioperative time course [PREOP and 2h after resection in the LV] (Mann‐Whitney *U* test) (B). *P < 0.05; **P < 0.005.

These so‐called M2 macrophages express high levels of CD163, which is cleaved and shed into the circulation. Accordingly, CD163 has been proposed as a surrogate parameter for an M2 phenotypical switch in macrophages.^(^ ^28^, ^29^ ^)^ In line with these findings, we observed a significant association of high sAXL and GAS‐6 concentrations and elevated sCD163, suggesting a pronounced M2 polarization (Fig. 4) Interestingly, however, patients with preoperatively high concentrations failed to increase circulating sCD163 immediately after induction of liver regeneration, suggesting a reduced shift in the M2 macrophage phenotype. Furthermore, a sparse increase postoperatively, as observed in patients with high preoperative concentrations, was associated with decreased soluble CD163 concentrations (fold change of AXL and fold change of sCD163: *R* = 0.616, *P* = 0.033 [Fig. 4B]); fold change of GAS‐6 and fold change of sCD163: *R* = 0.607], *P* = 0.036 [Fig. 4A]) (Fig. 4). In addition, MerTK, another receptor of the TAM‐RTK family, is prominently expressed on macrophages, including Kupffer cells. Similar to AXL, it is considered relevant for M2 polarization and sufficient regeneration after acute liver damage.^(^ ^24^ ^)^ Interestingly, no perioperative dynamics could be observed and overall concentrations were low, whereas for sAXL an increase could be noted (Fig. 4B). Accordingly, no significant association with sCD163 could be observed, neither with absolute concentrations nor with postoperative alterations (Fig. 4B). Overall, data obtained for GAS6 were most significant, possibly due to its solubility as well as affinity to AXL and MerTK.

As a limited M2 phenotypical switch of macrophages has been associated with pronounced inflammation, we further assessed pro‐inflammatory cytokine concentrations immediately after induction of liver regeneration.

### Elevated IL‐6 and CK18 as Indicators of Pronounced and Overwhelming Inflammation

**FIG. 5:**
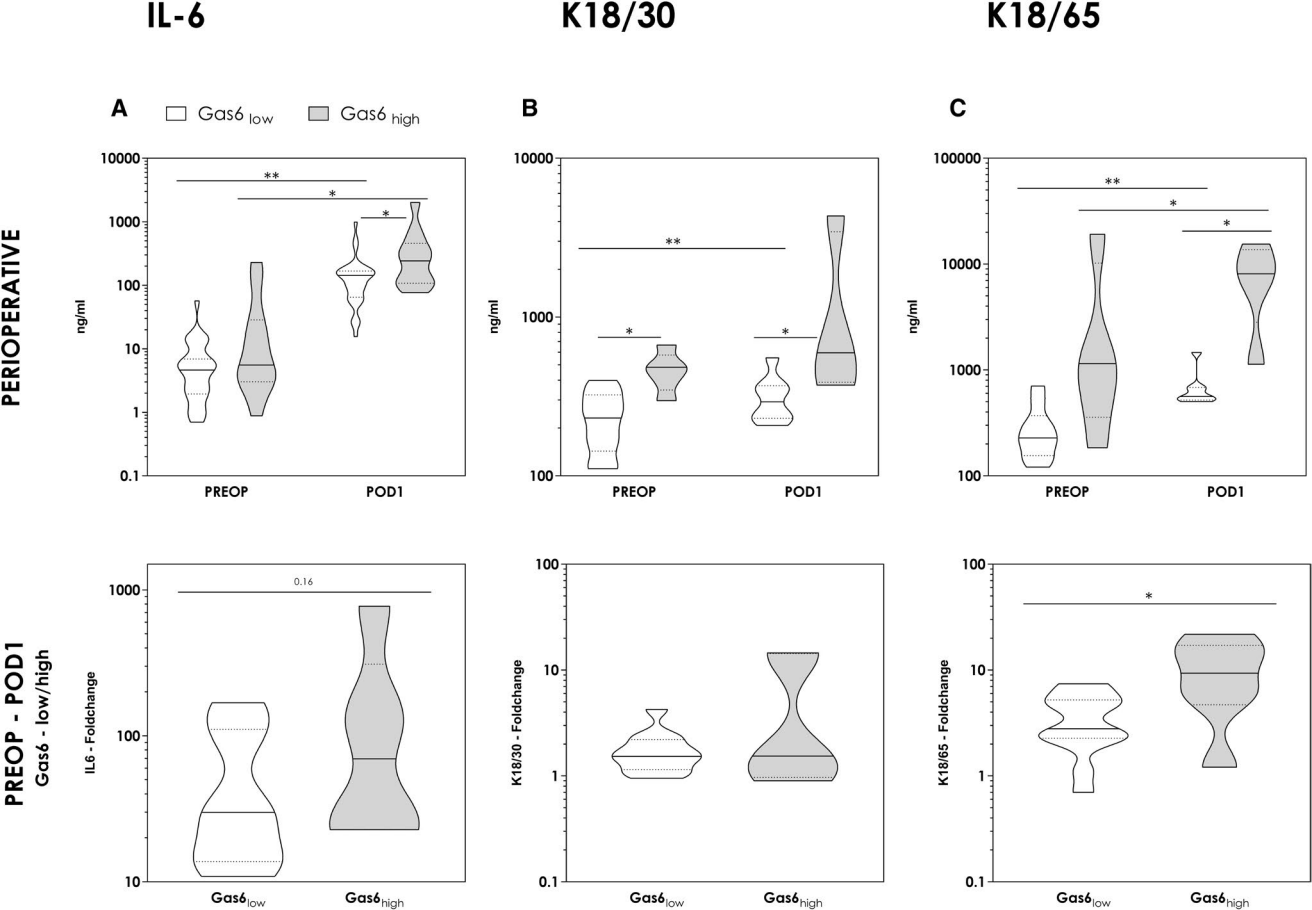
Pronounced postoperative inflammation and cellular demise in Gas6_high_ patients. To evaluate postoperative inflammation and resultant cell death, IL‐6 concentrations and cytokeratins were measured in the perioperative course. Results are displayed comparing Gas6_low_ and Gas6_high_ patients. (Mann‐Whitney U test and Wilcoxon signed‐rank test). For an exact evaluation of postoperative dynamics, observed alterations were evaluated and illustrated as fold change of IL‐6 as well as CK18/30 and CK18/65 (fold change = concentrations on POD1 divided by baseline concentrations [PREOP]) (Mann‐Whitney *U* test). *P < 0.05; **P < 0.005.

A phenotypical switch in favor of M2 polarization is also accompanied by a change in cytokine profile. Anti‐inflammatory cytokines are up‐regulated at the expense of pro‐inflammatory cytokines such as IL‐6. Accordingly, we observed a pronounced release of IL‐6 following liver resection and consequently also significantly higher circulating levels on POD1 in patients with preoperatively elevated GAS‐6 concentrations (Fig. 5A). Because extensive inflammation results in cellular demise, we further evaluated CK18 as a surrogate marker for intrahepatic cell death. To assess the proportional amount of necrosis and apoptosis, both the caspase‐cleaved CK18 (cCK18) by detection of the M30 epitope (CK18/30) and the full‐length protein CK18 were determined. Because a selective measurement of the uncleaved full‐length form is not possible, M5 and M6 epitopes (K18/65) are present on the caspase‐cleaved protein as well; the difference of CK18/65 and CK18/30 indicates the amount of necrosis. Both elevated CK18/30 and CK18/65 were associated with high GAS‐6 concentrations preoperatively as well as on POD1, and both rose significantly up to POD1 (Fig. 5B,C). However, contrary to CK18/30, we observed a significant difference in the postoperative increase of CK18/65, comparing patients with low and high preoperative GAS‐6 concentrations. High GAS‐6 concentrations before surgery and, consequently, a diminished GAS‐6 burst were associated with a significantly more pronounced release of CK18/65 (Fig. 5C). Hence, observed differences indicated elevated cell death in the form of necrosis under pronounced inflammatory conditions, as found in patients with insufficient GAS‐6 release following LR.

## Discussion

Within this study, we aimed to assess the relevance of the AXL/GAS‐6 pathway in liver surgery and regeneration. Initially, we found that patients with preoperatively increased sAXL and GAS‐6 levels suffered from postoperative LD and adverse clinical outcome significantly more frequently. Given the sensitive quantification of underlying liver disease, hepatic function and liver damage, these data provide strong exploratory evidence for their clinical suitability as biomarkers. However, even though patients suffering from LD were found to have elevated sAXL and GAS‐6 levels during the perioperative period, presumably caused by chronic activation via ongoing intrahepatic inflammation, these patients appeared to suffer from the exhaustion of this mechanism, as they failed to respond with an adequate “burst” immediately after the induction of liver regeneration. This rapid rise following liver resection, particularly regarding GAS‐6, appeared to be essential, as it was not only associated with intrahepatic immunomodulatory macrophage activation but also resulted in a reduction of pro‐inflammatory signaling during the early phase of liver regeneration. Therefore, our data provide distinct evidence for the critical relevance of this pathway in balancing inflammatory processes and counteracting overshooting intrahepatic inflammation during the priming phase of liver regeneration, as summarized in the graphical abstract.

Because adequate clinical management of insufficient regeneration and resulting LD is still limited, preoperative risk stratification is of critical importance to avoid this often fatal complication. Specifically in patients with underlying liver disease, markers to predict the ability of the remnant liver to regenerate after liver resection remain very limited. Only a few tests, invasive as well as noninvasive, were considered suitable for clinical application and hence have been clinically implemented. Limiting are especially cost efficiency, general availability, and invasiveness. Accordingly, circulating biomarkers appear to be an elegant solution.^(^ ^30^ ^)^ Considering low costs and easy applicability, an implementation is feasible and enables broad accessibility. In this context, sAXL and GAS‐6 are promising candidates. Recent studies demonstrated excellent predictive value regarding CLD.^(^ ^15^, ^16^, ^31^ ^)^ However, we report their promising predictive potential for postoperative LD in patients undergoing liver surgery. Accordingly, we found that elevated preoperative sAXL and GAS‐6 levels were directly associated with significantly worse clinical outcome after liver resection. Furthermore, both parameters are strong in prediction and (in terms of GAS‐6) independent from underlying liver disease (Table 3 [multivariate analysis] and Supporting Fig. S3). Their assessment hence represents a valuable tool for preoperative risk evaluation. However, while we observed rather close associations of sAXL and GAS‐6 with clinical outcome in a considerable amount of patients, prospective validation of these results is of utmost importance.

Kupffer cells release multiple cytokines and mediators, including TNF‐α, IL‐6 and IL‐1β, which have been shown to represent important inducers of liver regeneration.^(^ ^7^, ^32^ ^)^ Although these factors are certainly of importance as initial stimulus of liver regeneration, experimental models have also documented the necessity of tight regulation of these processes. For example, the simple duration of IL‐6 exposure was found to critically affect liver repair in mice, with adverse effects of prolonged exposure,^(^ ^33^ ^)^ but Kupffer cells do not simply exert pro‐inflammatory functions. The concept of M1 and M2 polarization has changed the understanding of tissue‐resident macrophages and Kupffer cells in particular. Multiple studies have now documented a very dynamic phenotypical plasticity. In addition to their pro‐inflammatory M1 phenotype, they further exhibit the capability to express anti‐inflammatory, pro‐regenerative cytokines, including IL‐10, IL‐13 or transforming growth factor beta (TGF‐β),^(^ ^7^, ^32^, ^34^, ^35^ ^)^ reflecting a phenotypical switch to an M2‐like state.^(^ ^21^, ^36^ ^)^ Tissue‐resident, as well as migrated macrophages, may therefore evidently account for the initial pro‐inflammatory stimulus of liver regeneration, while also tightly regulating intrahepatic inflammation. In this context, sCD163 represents a circulating monocyte/macrophage‐specific marker, which we found increased immediately after the induction of liver regeneration in our patients. As M2 macrophages express high levels of CD163,^(^ ^28^ ^)^ and the fact that CD163‐expressing macrophages are frequently found in areas of regenerating tissue after injury,^(^ ^37^ ^)^ its soluble form has been proposed as a marker, indicating macrophage phenotypical switch toward M2 polarization.^(^ ^38^, ^39^ ^)^ This is in line with a growing body of evidence suggesting that CD163 represents an anti‐inflammatory molecule.^(^ ^40^ ^)^

The regulation of this phenotypical switch is certainly multifactorial. However, TAM signaling (such as the AXL/GAS‐6 pathway) has been shown to critically affect this process as well as liver regeneration itself. Impaired TAM‐receptor surface cleavage in mice with bleomycin‐induced lung injury reduced inflammation and apoptosis, due to a shift in the cytokine profile. In particular, predominate expression of pro‐regenerative and anti‐inflammatory factors such as TGF‐β, as compared with pro‐inflammatory cytokines such as TNF‐α and IL‐1β, resembles a picture of a phenotypical macrophage switch to an M2‐like state.^(^ ^41^ ^)^ Similarly, experimental data associated absent TAM‐signaling after acute liver injury with a profound and overwhelming inflammatory response.^(^ ^23^, ^24^ ^)^ In this context, macrophage‐mediated immunomodulation might be of particular relevance. The anti‐inflammatory effect of GAS‐6 observed in a murine model for hepatic reperfusion/ischemia could be reproduced in a surrogate Kupffer cell line. Administration of GAS‐6 attenuated pro‐inflammatory stimuli, as TNF‐α and IL‐1β were distinctly down‐regulated by serine/threonine‐protein kinase–mediated prevention of nuclear factor kappa B (NF‐κβ) activation, shifting Kupffer cells from an M1 to an M2‐like phenotype.^(^ ^23^ ^)^ Similarly, other studies report on the GAS‐6‐mediated regulation of NF‐κB in macrophages.^(^ ^42^ ^)^ Furthermore, Rothlin et al. proposed AXL signaling as part of a counterregulatory feedback loop in response to pro‐inflammatory stimuli, as the initial signal transducer and activator of transcription (STAT)–mediated inflammatory response provokes an up‐regulation of AXL and hence TAM‐signaling.^(^ ^18^, ^43^ ^)^ Favoring an anti‐inflammatory macrophage phenotype, TAM signaling reduces pro‐inflammatory M1‐like macrophages as well as pro‐inflammatory cytokine expression.^(^ ^21^, ^36^, ^41^ ^)^ M2 macrophages further exert anti‐inflammatory and pro‐regenerative influence by the release of, for example, TGF‐β, IL‐4, and IL‐10.^(^ ^7^, ^32^, ^34^, ^35^ ^)^ These might, in turn, drive GAS‐6 release as reported by Nepal et al. and consequently sustain a balancing circuit in auto and paracrine manner.^(^ ^18^, ^44^ ^)^ In line with these results, GAS‐6‐knockout animals showed insufficient regeneration and more frequently succumbed to fulminant hepatic failure in a model of acute liver injury.^(^ ^23^ ^)^ Similarly, we observed a distinct dysregulation of the immediate induction during early liver regeneration in patients with preoperatively elevated GAS‐6 levels. In particular, an adequate uprise (“burst”) after induction of liver regeneration appeared to be of critical relevance for efficient liver regeneration in our patients. Despite sparse knowledge about TAM‐receptor shedding and its implications, it may be assumed that cellular up‐regulation translates into increased serum concentrations. Importantly, shedding occurs following activation.^(^ ^24^ ^)^ In accordance, Zagorska et al. reported prominent AXL activation following acute Jo2‐induced (Fas‐agonistic antibody‐mediated) liver injury in mice. Consequent cleavage was indicated by the appearance of its soluble domain 2 hours after Jo2 injection. Interestingly, MerTK was less affected, which might explain our results of overall low concentrations and the less pronounced postoperative dynamic (Fig. 4B). However, no exact conclusion regarding MerTK activation could be drawn from these experimental analyses, as MerTK knockout was detrimental in the context of liver injury, even though less pronounced than AXL knockout.^(^ ^24^ ^)^

In the context of TAM‐receptor signaling, M2 polarization might be of particular relevance. MerTK, which is strongly expressed on macrophages, including Kupffer cells, was previously linked to M2 polarization.^(^ ^24^, ^45^ ^)^ However, Kupffer cells, other than most resident macrophages, have also been shown to express high levels of AXL.^(^ ^24^ ^)^ As we did not observe any association of MerTK with CD163, whereas sAXL and GAS6 were found to correlate with postoperative CD163 dynamics (Fig. 4), the AXL/GAS6 axis might have increased relevance in M2‐macrophage switch as well as hepatic regeneration.^(^ ^24^ ^)^ However, the exact mechanism will have to be explored in consecutive experimental analyses. Furthermore, it has to be considered that also hepatic stellate cells, which are activated by TAM‐receptor signaling, especially via AXL/GAS6, might significantly contribute to hepatic regeneration. Following acute liver injury, hepatic stellate cell (HSC) activation is considered a physiological response mechanism promoting tissue repair. Concomitant GAS6 up‐regulation observed *in vitro*,^(^ ^46^ ^)^ as well as established TAM‐receptor up‐regulation in HSC,^(^ ^24^, ^46^ ^)^ might, in addition to Kupffer cells, account for the sAXL and GAS6 increase observed in patients undergoing liver resection as well as represent an additional critical mechanism of action of this pathway.

Intriguingly, patients with preoperatively elevated sAXL and GAS‐6 concentrations, appear to lack sufficient up‐regulation, suggesting exhaustion of this acute response in the context of CLD. Reduced liver regeneration in patients with underlying liver disease is clinically well established. However, mechanistic principles behind are only poorly understood, and existing rodent models of liver regeneration almost exclusively focus on healthy livers. Indeed, in the context of underlying liver disease, chronic inflammatory processes and, in particular, TAM signaling (such as the AXL/GAS‐6 pathway), might be of distinct relevance.^(^ ^47^ ^)^ Chronic exhaustive activation of this counterregulatory mechanism has been described in patients with CLD and might restain an additional increase upon acute liver injury and consequently regeneration.^(^ ^17^ ^)^ A loss of particular mechanism, however, is detrimental, as in several murine models with TAM receptor as well as ligand knockout following acute injury increased postoperative inflammation and cellular demise could be noted.^(^ ^23^, ^24^ ^)^

Consequently, we could demonstrate a significant association of sAXL as well as GAS‐6 expression and proinflammatory cytokine IL‐6. Along with an insufficient rise of sAXL and GAS‐6, increased IL‐6 expression could be noted (Fig. 5). These patients appeared to suffer from excessive immune activation following liver resection. The extent of TAM signaling may be inadequate to curb pro‐inflammatory processes to an optimal level, granting regeneration but avoiding intrahepatic cell damage. As we could demonstrate, this vacant resolution of postoperative inflammation, in the end, results in excessive cell demise. Significantly elevated levels of CK18 (intact full length) indicated pronounced cell death. CK18 is an intermediate filament important for epithelial and liver cells’ structural integrity, which is released upon cellular demise, and multiple previous reports indicated predictive value in terms of acute liver injury.^(^ ^48^, ^49^ ^)^ While its caspase‐cleaved form (cCK18) accumulates during apoptosis, the release of the full‐length protein is necrosis‐associated.^(^ ^50^ ^)^ cCK18 is measured selectively by detection of the M30 epitope, which is exposed by proteolytic cleavage during apoptosis. The two other common epitopes for detection are M6 and M5 (CK18/65). Present on both the full‐length protein and caspase‐cleaved fragment, these are postulated as a general indicator of cellular demise.^(^ ^49^, ^50^ ^)^ Interestingly, we could not observe a significant difference regarding the amount of apoptosis (CK18/30) comparing GAS‐6_low_ and GAS‐6_high_ cohorts, whereas CK18/65 concentrations rose significantly more in patients with high preoperative GAS‐6 levels. Consequently, these patients suffered from more intrahepatic cellular demise in general, considering the equivalent rise of apoptosis from pronounced nonapoptotic cell death, presumably necrosis.

In conclusion, this study provides distinct evidence for the relevance of AXL and GAS‐6 in hepatic immunomodulation, and thus for human liver regeneration. Preoperatively elevated levels were associated with preexisting liver disease, presumably caused by chronic intrahepatic inflammation. Consequently, we demonstrate the suitability of sAXL and GAS‐6 as predictive markers for the postoperative outcome, based on a large cohort of patients. Considering the advantages of blood‐prone biomarkers in terms of cost efficiency and accessibility, sAXL and GAS‐6 might represent a valuable tool for preoperative evaluation of liver function and regenerative reserve. If validated, a clinical implementation may help in the identification of patients at high risk, and thus provide a possibility to individualize treatment strategies.

In addition, we could demonstrate an immediate increase of sAXL and GAS‐6 following liver resection. However, patients who displayed an inadequate rise in sAXL or GAS‐6 were found to respond with overshooting intrahepatic inflammation. The gathered evidence suggests that the experimentally documented M1/M2 phenotypical switch of Kupffer cells is critical in this process. Given promising experimental treatment options for the manipulation of TAM‐receptor signaling, these translational data provide a central foundation for the development of therapeutic strategies also in humans. Because no therapeutic options are currently available for patients who develop postoperative LD, this might be of critical relevance.

## Supporting information

Fig S1

Fig S2

Fig S3

Fig S4

Supplementary Material
