# Supplementary figures and images for: Immunological Aspects of AXL/GAS‐6 in the Context of Human Liver Regeneration
Source: Hepatol Commun. 2021 Dec 24;6(3):576–92. doi: 10.1002/hep4.1832 (PMC8870037; doi:10.1002/hep4.1832)

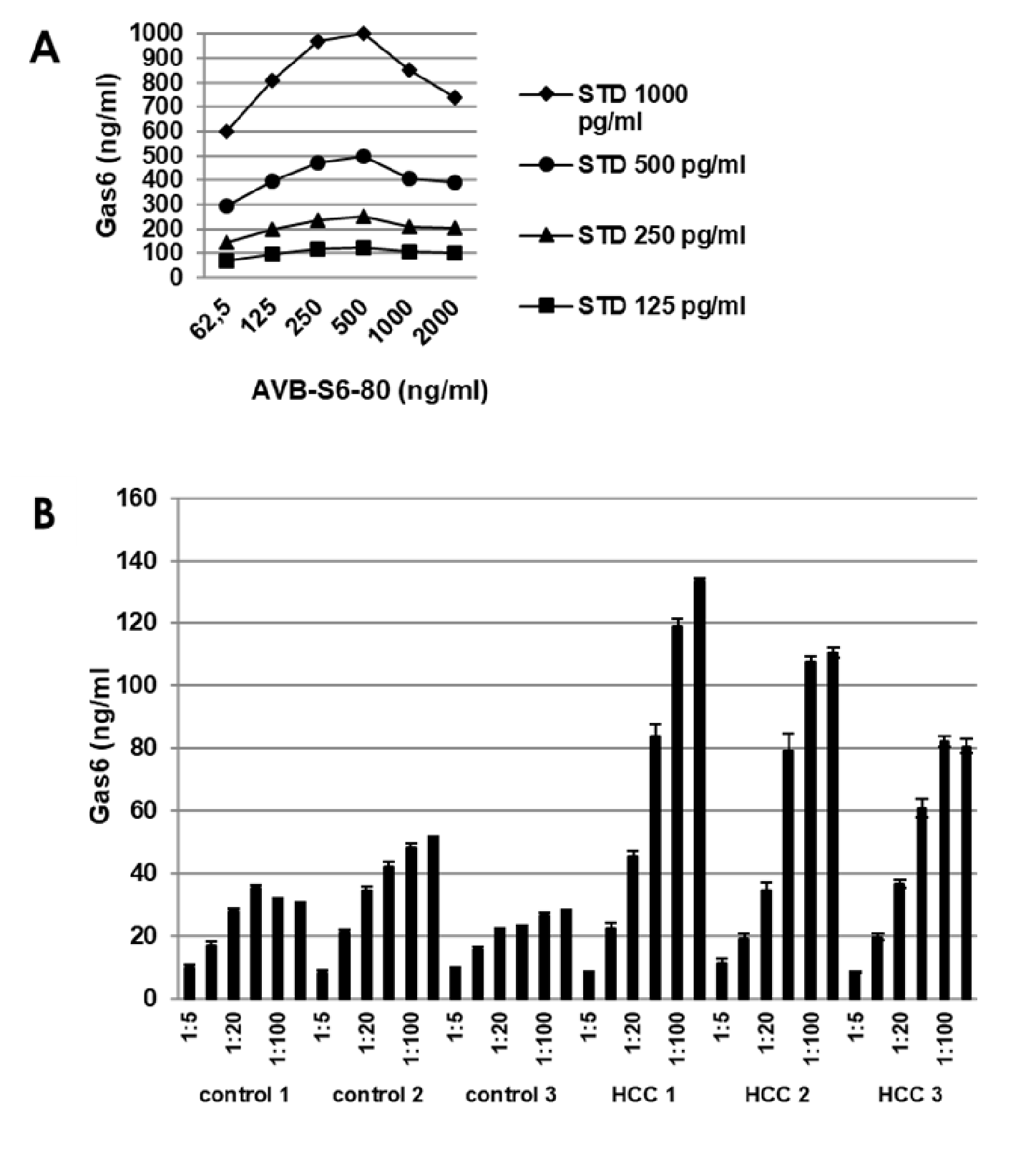

Supplement: Supplementary file 1 — Fig S1 [file HEP4-6-576-s002.tif]

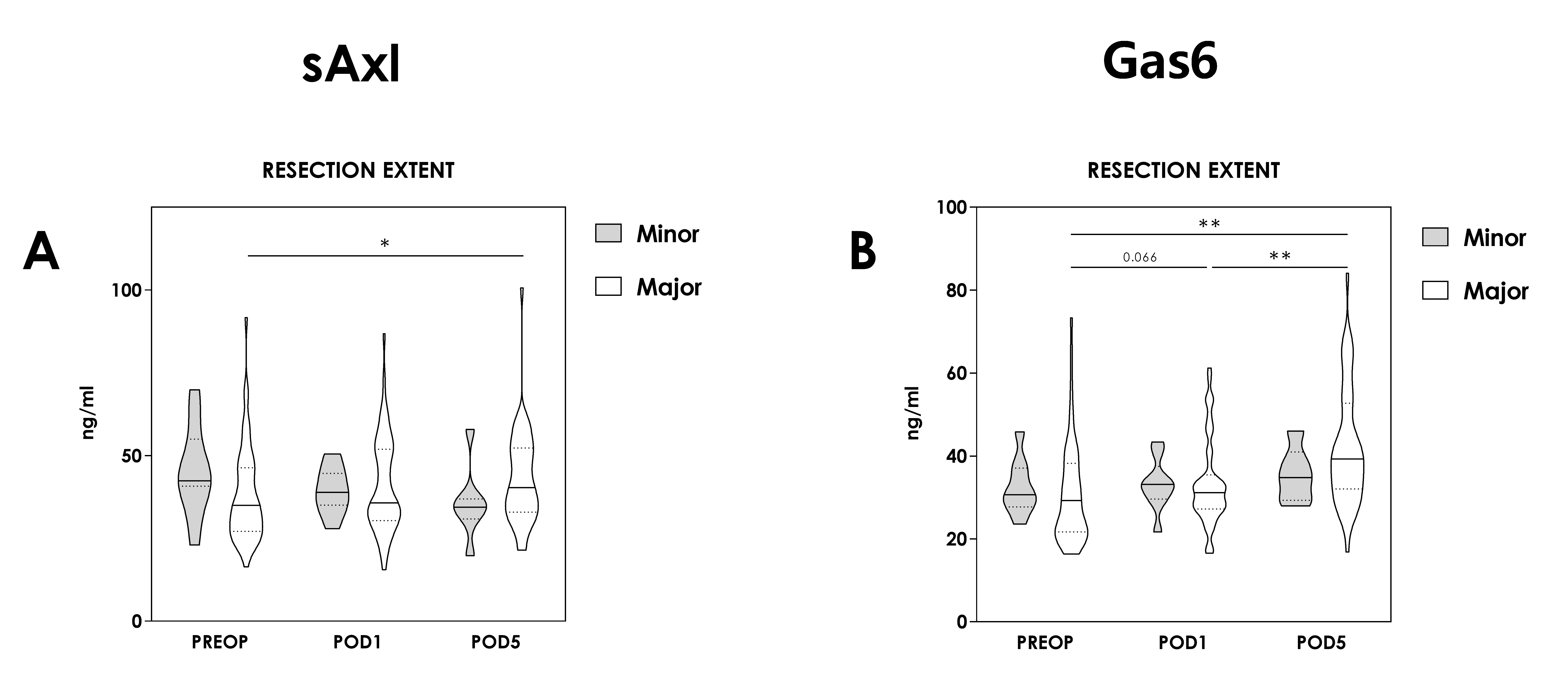

Supplement: Supplementary file 4 — Fig S4 [file HEP4-6-576-s005.tif]
