# Supplementary material for: Immunological Aspects of AXL/GAS‐6 in the Context of Human Liver Regeneration
Source: Hepatol Commun. 2021 Dec 24;6(3):576–92. doi: 10.1002/hep4.1832 (PMC8870037; doi:10.1002/hep4.1832)
Supplement: Supplementary file 5 — Supplementary Material [file HEP4-6-576-s003.docx]

**SUPPLEMENTARY DATA**

**Immunological aspects of AXL/GAS-6 in the context of human liver regeneration**

Gregor Ortmayr^1^, Laura Brunnthaler^2^, David Pereyra^1,2^, Heidemarie Huber^3^, Jonas Santol^1^, Benedikt Rumpf^1^, Sina Najarnia^1^, Rory Smoot^4^, Daphni Ammon^1^, Thomas Sorz^1^, Fabian Fritsch^1^, Michael Schodl^1^, Astrid Voill-Glaninger^5^, Barbara Weitmayr^6^, Manuela Födinger^7^, Martin Klimpfinger^8^, Thomas Gruenberger^9^, Alice Assinger^2^, Wolfgang Mikultis^3^, Patrick Starlinger^1,4^

^1^ Department of Surgery, Medical University of Vienna, General Hospital, Vienna, Austria

^2^ Center of Physiology and Pharmacology, Institute of Vascular Biology and Thrombosis Research,

Medical University of Vienna, Vienna, Austria

^3^ Department of Medicine I, Institute of Cancer Research, Comprehensive Cancer Center,

Medical University of Vienna, Austria.

^4^ Department of Surgery, Mayo Clinic, Rochester, MN, USA

^5^ Department of Laboratory Medicine, Viennese Health Network, Clinic Landstraße, Vienna, Austria

^6^ Department of Pathology, Viennese Health Network, Clinic Landstraße, Vienna, Austria

^7^ Department of Laboratory Medicine, Viennese Health Network, Clinic Favoriten, Vienna, Austria

^8^ Department of Pathology, Viennese Health Network, Clinic Favoriten, Vienna, Austria

^9^ Department of Surgery, HPB Center, Viennese Health Network, Clinic Favoriten and

Sigmund Freud Private University, Vienna, Austria

# Supporting Methods:

Definition of postoperative outcome
Postoperative outcome was prospectively documented and classified in LD, postoperative morbidity, and postoperative mortality. Liver dysfunction was assessed according to the ISGLS (International Study Group of Liver Surgery) criteria. (1) If patients were discharged because of favorable clinical development, prior to POD5, they were defined as no LD patients. The grade of postoperative morbidity was defined according to the Clavien-Dindo classification. Grade 3b and above were considered as severe morbidity. (2)

Liver vein sample acquisition:
Intraoperative samples were obtained (after parenchymal transection and ligation of the portal branch of the resected lobe) to evaluate alterations in the early phase of liver regeneration. Additional blood sampling consequently occurred from the remaining liver vein, draining the regenerating lobe as described by Starlinger et al. (3)

Enzyme-linked immunosorbent assay – sAXL/GAS
Serum levels of sAXL were detected by ELISA (human Axl DuoSet® ELISA (R&D Systems, Minneapolis, USA) as described recently. (4) In order to analyze Gas6 levels in patient sera, the human GAS-6 DuoSet® ELISAs (R&D Systems, Minneapolis, USA) was used under optimized assay conditions essentially as described for detecting sAXL. (5) Changes in the ELISA assay conditions included the replacement of GAS-6 capture antibody with AXL decoy receptor AVB-S6-80 (Aravive Biologics, Houston, Texas, USA), which captures GAS-6 with high affinity. (6) 500 ng/ml AVB-S6-80 were diluted in Dulbecco's phosphate-buffered saline (DPBS) and coated overnight to the microtiter plate at room temperature (Greiner Bio-One, Frickenhausen, Germany). Serum samples were analyzed at a dilution of 1:200 in LowCross-buffer^®^ (Candor Bioscience, Wangen, Germany). The GAS-6 detection antibody was diluted in DPBS. (Fig. S1)

Enzyme-linked immunosorbent assay – sMERTK163
**(Invitrogen - Mer (MERTK) Human ELISA Kit)**The assay was performed according to the manufacturer's instructions.

Enzyme-linked immunosorbent assay – sCD163  **(R&D systems - Human CD163 Quantikine ELISA Kit)**The assay was performed according to the manufacturer's instructions.

Enzyme-linked immunosorbent assay - Interleukin 6  **(R&D systems - Human IL-6 Quantikine ELISA** **Kit**)
The assay was performed according to the manufacturer's instructions.

#

# Supporting Figures:


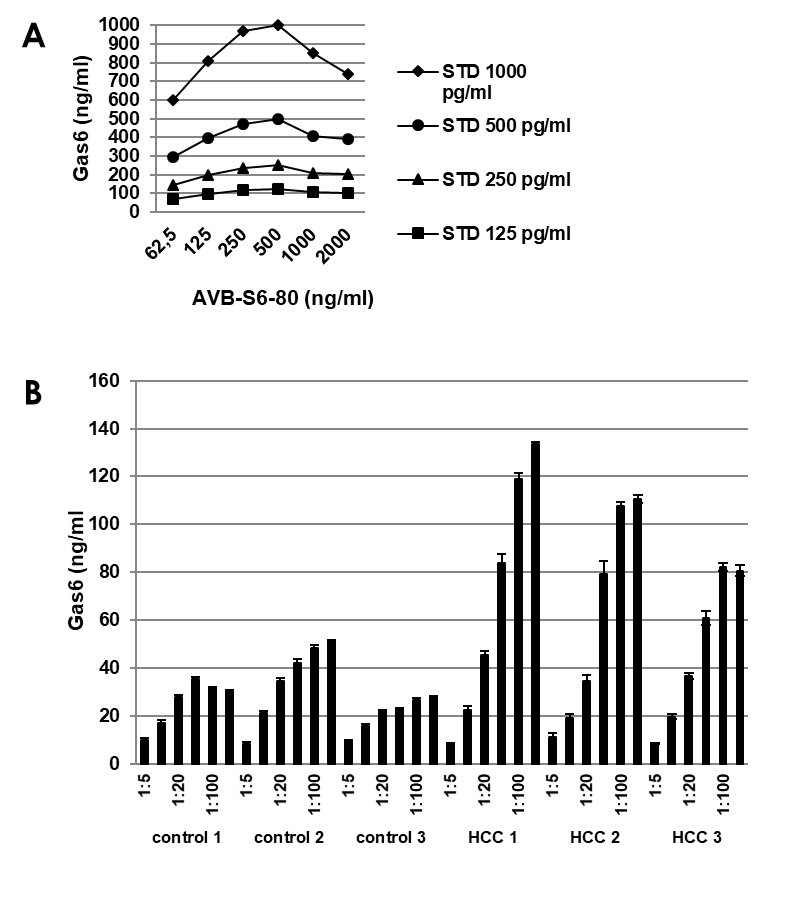


**Fig. S1
Optimization of Axl decoy receptor concentrations and sample dilutions for detecting Gas6 in serum samples by ELISA.**
(A) Microtiter plates were coated with different concentrations of the Axl decoy receptor AVB-S6-80 and analyzed with recombinant Gas6 standards (STD). (B) Serum samples of healthy volunteers (control 1-3) and 3 HCC patients were diluted with LowCross-buffer® in a range from 1:5 to 1:200 and analyzed for GAS-6 levels by ELISA. Data points represent mean ± standard deviation (SD) of triplicate wells (n = 3).


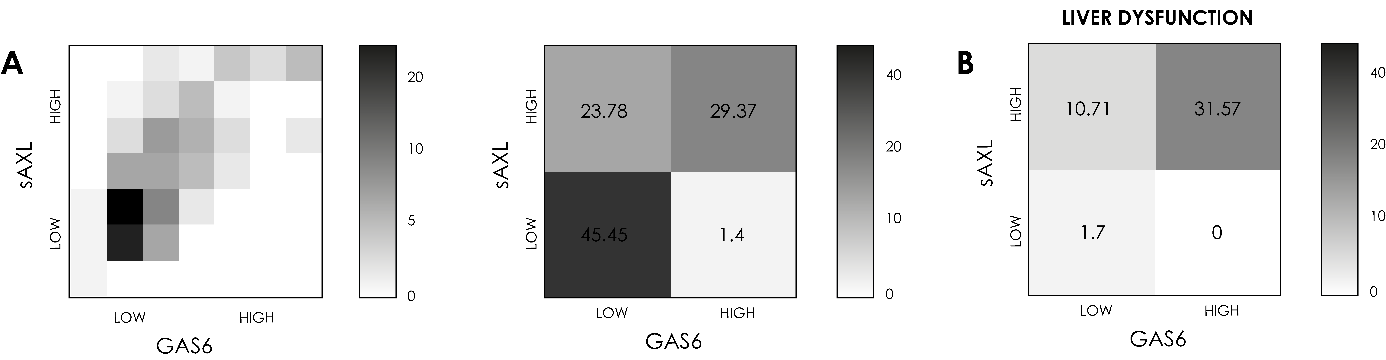


**Fig. S2
Association of sAxl, Gas6 and LD**(A) Correlation is visualized as distribution of patients according to their preoperative sAxl and Gas6 concentrations. (B) To further indicate the association with LD, the illustration in (B) is supplemented by the aspect of LD. Coloring corresponds to the relative incidence.

**
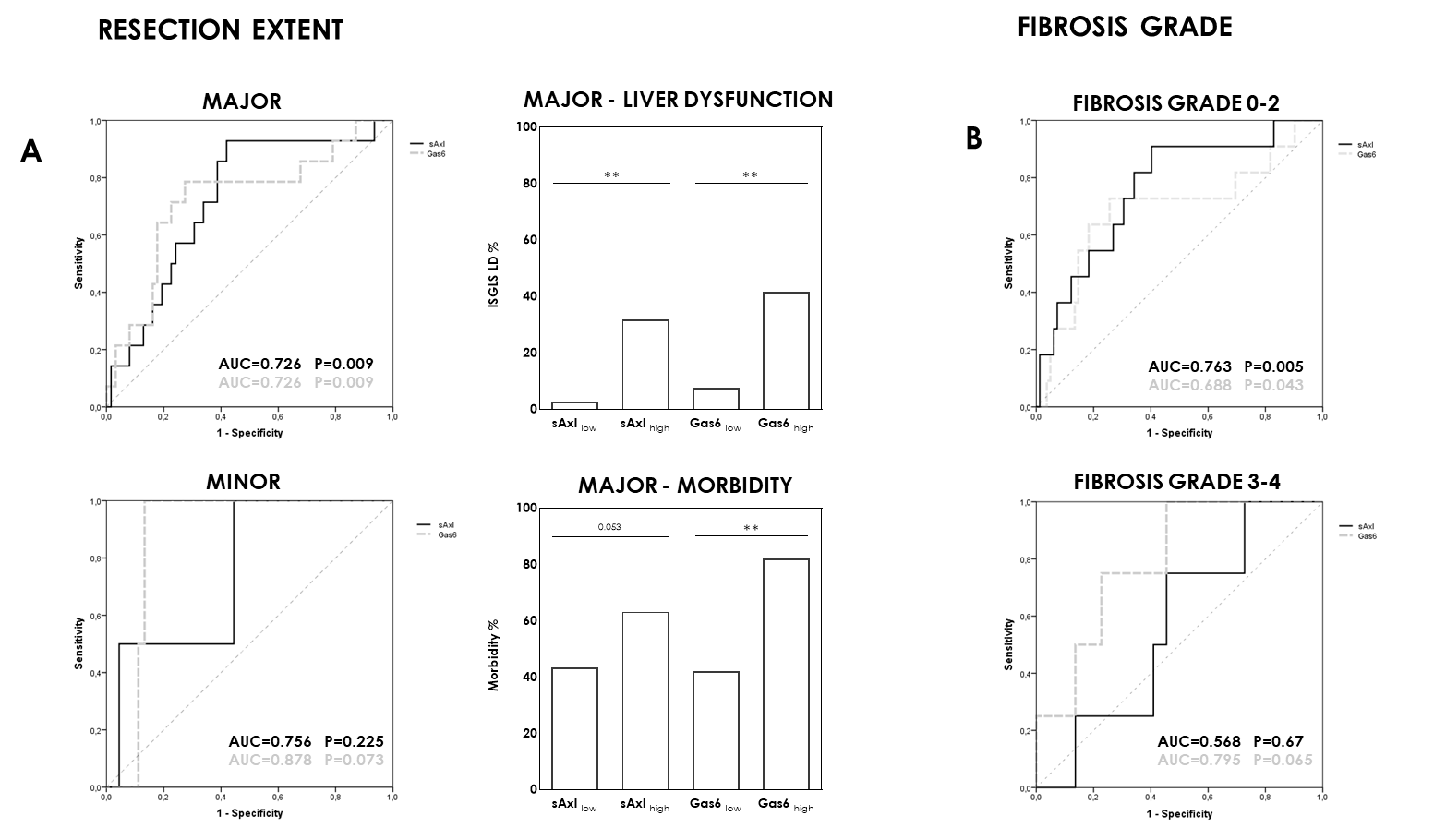
**

**Fig. S3
sAxl and Gas6 – prediction of postoperative outcome in dependency to resection extent and fibrosis**

To evaluate the influence of resection extent and grade of fibrosis on the prediction of postoperative outcome, ROC analysis was performed comparing major and minor resection (A) as well as low (0-II) and advanced fibrosis grade (III-IV) (B). Risk stratification of patients according to chosen cut-offs visualizes the variance of incidence among low and high-risk groups for postoperative LD and morbidity undergoing either major or minor resection [chi-squared test] (A). According to the IHPBA‐Brisbane‐2000 nomenclature, minor was classified as <3 segments, major resections as ≥3 segments; Morbidity and severe Morbidity were assessed according to the Clavien-Dindo classification; LD according to the ISGLS-criteria (International Study Group of Liver Surgery). *P < 0.05; **P < 0.005


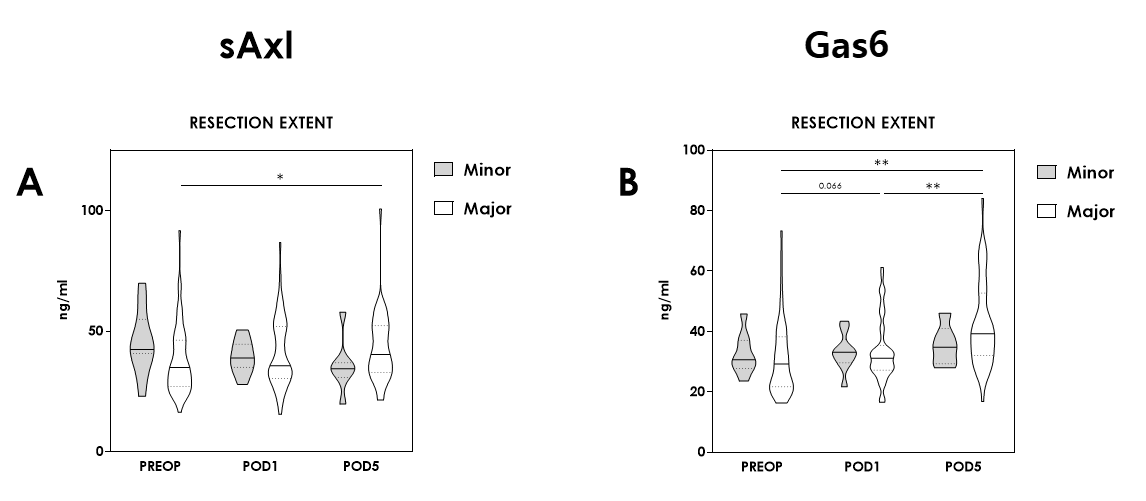


**Fig. S4
Perioperative time course dependent on resection extent**

sAxl (A) and Gas6 (B) concentrations were measured preoperatively (PREOP), on postoperative day 1 (POD1) and day 5 (POD5). Perioperative dynamic is illustrated comparing major and minor resection [Mann‐Whitney U test and Wilcoxon Sign test]. According to the IHPBA‐Brisbane‐2000 nomenclature, minor was classified as <3 segments, major resections as ≥3 segments. *P < 0.05; **P < 0.005

| **Table S1 -** **Collinearity diagnostics** | | | | | | | | | |
| --- | --- | --- | --- | --- | --- | --- | --- | --- | --- |
|  | **Model** | | | **Collinearity  statistics** | | | | **Model comparison** | |
| **Parameter** | **OR** | **95%CI** | **p-value** | **Tolerance** | **VIF** | **Eigenvalue (lowest)** | **Variance Proportion** | $x$^2^ **(p-value)** | |
| **sAxl (ng/mL)** | 1.049 | 1.013 – 1.086 | 0.007 |  |  |  |  | 0.371 (p=0.543) |  |
| **Gas6 (ng/mL)** | 1.049 | 1.012 – 1.088 | 0.010 |  |  |  |  |  | 0.188 (p=0.17) |
| **sAxl (ng/mL)** | 1.036 | 0.985 | 0.171 | 0.453 | 2.208 | 0.035 | 0.88 |  |  |
| **Gas6 (ng/mL)** | 1.017 | 0.964 | 0.538 | 0.453 | 2.208 | 0.035 | 0.86 |  |  |
| OR = odds ratio, 95%CI = 95% confidence interval | | | | | | | | | |

**UNIVARIATE**

**MULTIVARIATE**

# References:

1. Rahbari NN, Garden OJ, Padbury R, Brooke-Smith M, Crawford M, Adam R, et al. Posthepatectomy liver failure: a definition and grading by the International Study Group of Liver Surgery (ISGLS). Surgery. 2011;149(5):713-24.

2. Dindo D, Demartines N, Clavien P-A. Classification of surgical complications: a new proposal with evaluation in a cohort of 6336 patients and results of a survey. Annals of surgery. 2004;240(2):205-13.

3. Starlinger P, Pereyra D, Haegele S, Braeuer P, Oehlberger L, Primavesi F, et al. Perioperative von Willebrand factor dynamics are associated with liver regeneration and predict outcome after liver resection. Hepatology (Baltimore, Md). 2018;67(4):1516-30.

4. Dengler M, Staufer K, Huber H, Stauber R, Bantel H, Weiss KH, et al. Soluble Axl is an accurate biomarker of cirrhosis and hepatocellular carcinoma development: results from a large scale multicenter analysis. Oncotarget. 2017;8(28).

5. Dengler M, Huber H, Müller CJ, Zellmer A, Rauch P, Mikulits W. Accurate Determination of Soluble Axl by Enzyme-Linked Immunosorbent Assay. Assay and drug development technologies. 2016;14(9):543-50.

6. Kariolis MS, Miao YR, Jones DS, 2nd, Kapur S, Mathews, II, Giaccia AJ, et al. An engineered Axl 'decoy receptor' effectively silences the Gas6-Axl signaling axis. Nature chemical biology. 2014;10(11):977-83.
